# Long Term Administration of Nivolumab for Metastatic Melanoma: A Case Report

**DOI:** 10.7759/cureus.26359

**Published:** 2022-06-27

**Authors:** Adela-Raluca Oprea, Arnaud Benas, Andrei Havasi, Alecsandra Gorzo, Stefan Spinu, Daniel Sur, Claudia Burz

**Affiliations:** 1 Oncology, Oncology Institute "Prof.Dr.Ion Chiricuta" Cluj-Napoca, Cluj-Napoca, ROU; 2 Oncology, Iuliu Hațieganu University of Medicine and Pharmacy, Cluj-Napoca, ROU

**Keywords:** administration, nivolumab, immunotherapy, metastatic, melanoma

## Abstract

The treatment of metastatic melanoma changed dramatically with the discovery of immune checkpoint inhibitors (ICIs). Patients face prolonged exposure to these agents, which can frequently generate a large spectrum of adverse reactions. It has been shown that a considerable number of patients treated with ICIs achieve a durable response to treatment that is maintained even after cessation. We present the case of a 75-year-old man with metastatic melanoma who underwent 95 cycles of nivolumab without significant treatment-related toxicities or progression. Future studies are needed to deﬁne more clearly the optimal duration of anti-programmed cell death protein 1 (PD-1) agents in patients with good tolerance and no progression. Patients could avoid discomfort caused by frequent physician visits, high additional costs, and possible adverse reactions that may occur after such a long period of exposure to immunotherapy.

## Introduction

Melanoma is an aggressive and lethal tumor caused by the malignant transformation of melanocytes with an increased capacity for lymphatic and hematogenous metastasis [1]. Although mortality rates have been declining in recent years [2], the incidence of melanoma continues to rise with 324,635 new cases globally in 2020 [3]. The five-year relative survival rate is 99% for stage I and drops to 27% in metastatic disease [2]. Most cases are sporadic, and various genetic mutations are involved in melanoma development, with the somatic activating mutations in the proto-oncogene B-Raf and v-Raf murine sarcoma viral oncogene homolog B (BRAF) gene (BRAF V600E) remaining the most significant ones [4].

The management of patients with metastatic melanoma is fundamentally based on a systemic treatment that may be complemented in selected patients by surgical treatment and radiotherapy. Until recently, the only systemic therapy available to these patients was chemotherapy with dacarbazine, alkylating agents, or taxanes [5]. Nowadays, therapeutic options are represented by immune checkpoint inhibitors (ICIs) such as cytotoxic T-lymphocyte-associated protein 4 (CTLA-4) inhibitors (ipilimumab), programmed cell death protein 1 (PD-1) inhibitors (nivolumab or pembrolizumab), and targeted therapies with BRAF and mitogen-activated extracellular signal-regulated kinase (MEK) inhibitors [6]. Nivolumab is a fully-humanized anti-PD-1 monoclonal antibody that selectively blocks the interaction of PD-1 with its receptors BRAF and programmed death-ligand 2 (PD-L2) and restores T-cell response directed at tumor cells, inducing an anti-tumor eﬀect [7]. Since 2015 it has been approved for the treatment of metastatic melanoma in patients without BRAF mutation regardless of programmed death-ligand 1 (PD-L1) expression proving superiority in terms of overall survival compared to chemotherapy with dacarbazine [8]. The most common adverse effects encountered in nivolumab monotherapy are related to autoimmunity, including colitis, pneumonitis, hepatitis, increased amylase, lipase, or dermatological toxicities [9].

In this report, we describe a case of a male patient with advanced locoregional melanoma, initially treated surgically and with adjuvant interferon, subsequently metastasized, who underwent 95 cycles of nivolumab without major treatment-related toxicities or progression. Although the nivolumab product label recommends pursuing treatment until signs of disease progression or unacceptable toxicity appear, further studies focused on nivolumab treatment duration are necessary.

We present this case as per the case reports (CARE) checklist.

## Case presentation

A 75-year-old man with a previous history of non-insulin-dependent type 2 diabetes mellitus, primary arterial hypertension, obesity, and venous ulcer of the lower extremities, presented to our clinic in September 2015 with a recent diagnosis of nodular malignant melanoma on the left paravertebral area, ulcerated, Clark level III, Breslow thickness 18 mm. The patient had undergone an incomplete excision in another hospital, and he presented to our surgery department to complete the surgical sequence. Physical examination revealed the presence of left axillary lymphadenopathy measuring approximately 60/50 mm, which was subsequently resected through lymphadenectomy level 1-2 Berg, along with re-excision of the skin tumor. The histopathological report revealed axillary lymph node metastasis from melanoma (1/15) and cutaneous excision piece without tumor cells. The disease stage was classified as pT4bN1M0 stage III C (AJCC 7th edition). Adjuvant interferon alfa-2b treatment was initiated in December 2015 at a dose of 9 mg/day, three days per week, and was administered for one year with good overall tolerance. In February 2017, a follow-up contrast-enhanced chest-abdomen-pelvis computed tomography (CAP-CT) scan revealed the presence of a 28 mm, cT1aN0M0, stage I tumor in the right kidney (American Joint Committee on Cancer (AJCC) 7th edition). Following right nephrectomy, histopathological examination described the presence of clear cell renal carcinoma, Fuhrman grade 2, delimited by a fibrous pseudocapsule without invasion. Given the stage of the disease, we did not institute adjuvant treatment.

**Figure 1:**
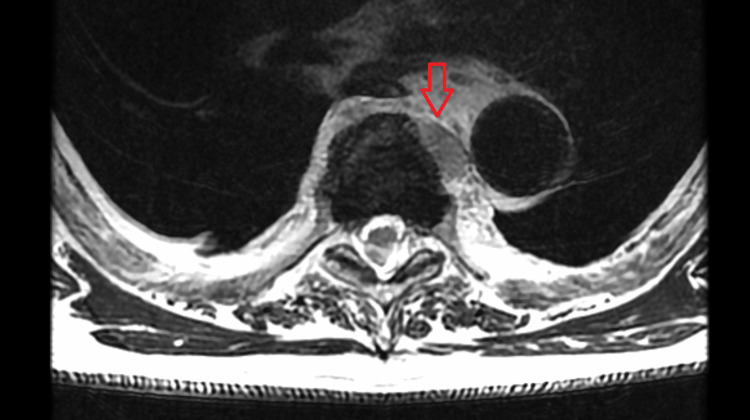
MRI of the dorso-lumbar spine showing a tumor mass in the T5 vertebra

In November 2017, the patient presented with cervical and dorso-lumbar pain and motor deficit. A non-contrast brain and cervical spine CT scan was performed, revealing an area of bone lysis at the C1 vertebra. Contrast-enhanced magnetic resonance imaging (MRI) of the brain and cervical spine confirmed the presence of a tumor lesion in the C1 vertebra with extension to adjacent soft tissues and identified possible tumor involvement of the T5 vertebra. An MRI of the dorso-lumbar spine identified the presence of tumor lesions in the T5 and S1 vertebra, pathological compression of the T5 vertebra, and a nodular lesion in the right iliac bone measuring approximately 20 mm. The neurosurgical consult did not indicate surgery (Figure 1). 

At the end of 2017, the patient was hospitalized for diagnosis confirmation. Given the presence of the two oncological pathologies, a biopsy of the dorso-lumbar spine was performed, which established that the bone lesions were metastases of melanoma, BRAF negative. Therefore, in January 2018, we decided to initiate monotherapy with nivolumab 240 mg every two weeks, and zoledronic acid 4 mg every four weeks.

After six doses of nivolumab, the pain was controlled, and the patient recovered from the motor deficit. Follow-up contrast-enhanced CAP-CT revealed the presence of a newly arising 10 mm nodular lesion in the left axillary subcutaneous tissue and several subcentimeter lymph nodes at the para-aortic, bilateral external iliac, and bilateral inguinal levels. Pelvic bone metastases increased in size and number compared to the previous scan, and vertebral bone metastases persisted. Due to improvement in symptomatology, it was interpreted as partial remission.

Nivolumab treatment continued until September 2018, when the patient presented to the emergency room for an episode of loss of consciousness. A CT scan of the brain was performed, which showed no abnormal changes. A blood workup revealed grade 2 hepatocytolysis and cholestasis. Infectious etiology of disease progression was ruled out as the cause for liver function impairment. We concluded that the hepatic cytolysis syndrome occurred as toxicity due to nivolumab treatment. And so corticosteroid treatment was started, and nivolumab administration was delayed. After two weeks, blood tests improved, and treatment was resumed.

By January 2022, the patient had completed 95 cycles of nivolumab without significant laboratory test changes or important side effects. Except for the development of new bone metastases on the costal arches, the follow-up CT scans performed at regular intervals showed stable disease.

All procedures performed in this study were in accordance with the standards of ethical research and written informed consent was obtained from the patient for publication of this case report and accompanying images. 

## Discussion

In recent years, the discovery of the critical role of the PD-1 pathway in the treatment of melanoma has led to a considerable improvement in the prognosis of patients facing this pathology. According to guidelines at the time of initiation of therapy, interferon alfa-2b was commonly used as adjuvant treatment in stage III C melanoma, after surgical resection [10].

An important aspect of this report is the presence of clear cell renal carcinoma. Several studies have confirmed the bidirectional association between malignant melanoma and renal cell carcinoma [11]. Thus, it has been shown that patients with malignant melanoma who develop renal cell carcinoma during their disease are more likely to be elderly, male, and Caucasian [11]. In terms of tumor histology and tumor, nodes, and metastases (TNM) staging, clear cell type, and stage pT1a, are more common, as in our case [11,12]. Although several genetic abnormalities are associated with both cancer types, no genetic tests were performed in the present case [13].

Approximately 10 months after cessation of interferon treatment, follow-up imaging investigations revealed the presence of several melanoma bone metastases, which were subsequently confirmed on histopathological examination. In the absence of BRAF mutation, first-line therapy of metastatic melanoma is limited to two options: monotherapy with anti-PD-1 agents (nivolumab or pembrolizumab) or a combination of an anti-PD-1 agent and an anti-CTLA-4 agent (nivolumab and ipilimumab) [6]. The results of the CheckMate-067 trial showed the superiority of dual blockade over nivolumab or ipilimumab monotherapy in terms of progression-free survival (PFS). Comparing nivolumab and ipilimumab combination with nivolumab monotherapy, differences in overall survival (OS) were insignificant. In addition, the combination of the two agents was shown to result in higher immune-mediated toxicity compared to nivolumab monotherapy [14]. Thus, given the patient's age, Eastern Cooperative Oncology Group (ECOG) performance status (PS)= 2, uncontrolled diabetes and hypertension, and increased risk of toxicity caused by the ipilimumab and nivolumab combination, we decided to initiate immunotherapy with nivolumab 240 mg every two weeks.

Studies have shown that liver toxicity caused by nivolumab treatment is a relatively low-frequency event [8,15], the time interval between treatment initiation and the occurrence of hepatotoxicity being approximately 14 weeks [16]. Management of nivolumab-induced hepatotoxicity frequently involves the administration of corticosteroids. In patients with moderate hepatic impairment, also present in this report, guidelines recommend assessing liver function every three to five days and deferring immunotherapy until aspartate transaminase (AST) and alanine transaminase (ALT) values normalize [9].

Identifying predictive markers of response to immunotherapy is a key topic for current research. Regarding serum lactate dehydrogenase (187 U/L) and C-reactive protein (0.263 mg/dl) levels at baseline, they were within normal limits. The absolute lymphocyte count at six weeks after initiation of nivolumab treatment was 1520/μl, neutrophil/lymphocyte ratio at baseline was 6.9. Since low or negative PD-L1 expression does not exclude a response to nivolumab immunotherapy, we did not perform determinations in this regard. As pretreatment prognostic factors, elevated serum lactate dehydrogenase and C-reactive protein levels at baseline are associated with poorer response to immunotherapy and lower overall survival (OS) [17]. Leukocyte subtype counts have also been studied as a possible biomarker capable of predicting response to ICIs treatment. An absolute lymphocyte count >1000/μl at three to six weeks after initiation of nivolumab therapy is associated with better OS. In terms of neutrophil/lymphocyte ratio, an initial value higher than the cut-off value is related to lower OS [17,18]. Although many studies have investigated the utility of measuring PD-L1 expression as a predictor of response to anti-PD-1 agents, it has been concluded that, in melanoma patients, the low or negative expression does not exclude a response to this type of immunotherapy [14,15]. Another possible predictor of response to PD-1 inhibitor immunotherapy is peritumoral inflammatory infiltrate. In patients with melanoma, a peritumoral and intratumoral cluster of differentiation 8 (CD8)+ T-cell density has been found to have a predictive value for treatment response [19].

Trials leading to the approval of PD-1 inhibitor immunotherapy for patients with metastatic melanoma support a treatment duration of two years or until progression or unacceptable toxicity occurs [20,21]. For nivolumab treatment, the median time from initiation of therapy to objective response is approximately 10 weeks [8,14,15]. In the present case, to date, the best objective response of target lesions obtained according to response evaluation criteria in solid tumors (RECIST) 1.1 criteria has been stable disease (SD), with no clear evidence of progressive disease on follow-up imaging investigations. Regarding the events occurring during immunotherapy, we can state that it was well tolerated, without major treatment-related toxicities. Thus, the most important question of this report comes in: when should we stop nivolumab administration?

Trials including patients treated with nivolumab, have shown that the most common reasons given for discontinuation were the occurrence of signs of disease progression or high-grade toxicity [8,15]. In addition, despite treatment cessation, a response can be maintained over a long period [22]. Available data indicate that even patients who discontinued treatment for other reasons than progression or toxicity maintained a response for 16 to 56 weeks [23]. A study evaluating the response of 185 patients to discontinuation of anti-PD-1 agents in the absence of progression or toxicity showed that the risk of disease progression after discontinuation was higher in patients whose initial objective tumor response was SD (50%) compared to those who initially experienced a complete response (CR) to treatment (14%). In addition, when a rechallenge with the same agent was attempted, a proportion of patients with progression experienced a new response to treatment. The same study found that of all patients who achieved a CR to therapy, those who received treatment for less than six months had a higher risk of progression [24]. Based on this evidence, there may be an inverse relationship between duration and initial response to treatment and the subsequent risk of disease progression. The current trend in patients with complete or partial response (PR) to immunotherapy with anti-PD-1 agents is to shorten as much as possible the period of administration to minimize toxicities but still achieve a maximum anti-tumor effect.

The Keynote-001 study showed that 61 of 67 patients who achieved a CR following pembrolizumab therapy and electively discontinued treatment after an average of 23 months maintained their response to treatment after 24 months of follow-up [20]. The Keynote-006 trial comparing pembrolizumab versus ipilimumab immunotherapy set a maximum pembrolizumab administration period of two years. Also, after a minimum of six months of pembrolizumab administration, patients who achieved a CR on therapy were allowed to discontinue treatment after two more cycles. From the total number of patients treated with this agent, only 103 completed two years of treatment. Final findings showed that both patients treated for two years and those treated for a minimum of six months with pembrolizumab had similar PFS outcomes at 24 months [21]. In contradiction, the CheckMate-153 trial which included patients with non-small cell lung cancer (NSCLC) compared two arms of nivolumab administration: one-year versus continuous until progression or unacceptable toxicity occurred. In patients with CR and PR, both median PFS and median OS were higher in the continuous treatment arm compared with the arm that discontinued treatment after one year. Interestingly, patients with SD had similar median PFS and OS results in both arms [25].

The main argument for limiting the administration period is to avoid possible toxicities, especially late-onset ones. Most immune-related adverse events (irAEs) generally occur early, in the first months after the initiation of immunotherapy [23]. In treatment with anti-PD-1 inhibitors, with or without CTLA-4 inhibitors, the incidence of late-onset irAEs (>1 year) is higher in patients treated for metastatic disease. Anti-PD-1 monotherapy seems to generate more frequent late toxicity compared to combination therapy. Although there have been cases in which these late events occurred after cessation of treatment, most irAEs occurred in patients still on treatment. Among patients with metastatic disease, it appears that patients with CR have a higher risk of developing late irAEs than those with SD [26]. A study analyzed the probability of late-onset irAEs in melanoma and lung cancer patients treated with ICIs [27]. At 24 months after initiation of therapy, the probability of developing an irAE was 57.3%. Comparative data of different types of ICIs identified anti-PD-1 agents as having the best safety profile for late-onset irAEs.

Although a shorter duration of treatment may decrease the risk of late-onset irAEs, the decision to discontinue therapy should be carefully weighed, as these have been shown to occur even after immunotherapy is stopped.

## Conclusions

In this report, we dealt with a patient with SD who was at increased risk of progression if immunotherapy is ceased. We consider that avoiding potential late-onset irAEs should not be an argument for stopping a still effective treatment. Therefore, we emphasize once again the need for future studies to define more clearly the optimal duration of administration of anti-PD-1 agents in patients with good tolerance and no progression under treatment.
